# Mitochondrial-nuclear coadaptation revealed through mtDNA replacements in *Saccharomyces cerevisiae*

**DOI:** 10.1186/s12862-020-01685-6

**Published:** 2020-09-25

**Authors:** Tuc H. M. Nguyen, Sargunvir Sondhi, Andrew Ziesel, Swati Paliwal, Heather L. Fiumera

**Affiliations:** 1 Department of Biological Sciences, Binghamton University, Binghamton, NY USA; 2 Department of Bioscience and Biotechnology, Banasthali Vidyapith, Rajasthan, India

**Keywords:** Mitonuclear, Cytonuclear incompatibilities, Mt-n, Mitochondrial-nuclear, Coevolution, Coadaptation, G × G, G × G × E, *Saccharomyces*

## Abstract

**Background:**

Mitochondrial function requires numerous genetic interactions between mitochondrial- and nuclear- encoded genes. While selection for optimal mitonuclear interactions should result in coevolution between both genomes, evidence for mitonuclear coadaptation is challenging to document. Genetic models where mitonuclear interactions can be explored are needed.

**Results:**

We systematically exchanged mtDNAs between 15 *Saccharomyces cerevisiae* isolates from a variety of ecological niches to create 225 unique mitochondrial-nuclear genotypes. Analysis of phenotypic profiles confirmed that environmentally-sensitive interactions between mitochondrial and nuclear genotype contributed to growth differences. Exchanges of mtDNAs between strains of the same or different clades were just as likely to demonstrate mitonuclear epistasis although epistatic effect sizes increased with genetic distances. Strains with their original mtDNAs were more fit than strains with synthetic mitonuclear combinations when grown in media that resembled isolation habitats.

**Conclusions:**

This study shows that natural variation in mitonuclear interactions contributes to fitness landscapes. Multiple examples of coadapted mitochondrial-nuclear genotypes suggest that selection for mitonuclear interactions may play a role in helping yeasts adapt to novel environments and promote coevolution.

## Background

Mitochondria are the cytoplasmic organelles that power eukaryotic life. Their functions in energy production, nutrient and redox sensing and signaling pathways are governed by both the mitochondrial and nuclear genomes. Known genetic interactions between these distinct organellar DNAs are required for basic mitochondrial functions including mitochondrial DNA (mtDNA) replication and repair, transcription and translation of mitochondrial genes, assembly and function of the respiratory chain complexes and creation of a mitochondrial membrane potential that drives energy production and maintains mitochondrial homeostasis [1, 2]. These genetic interactions have shaped the evolution of both genomes [3–5].

As mtDNAs diverge in different populations of a species, selection should favor coevolved nuclear alleles that help maintain important mitochondrial activities. During hybridization, these coevolved mitochondrial-nuclear (mitonuclear) allele pairs may be disrupted resulting in reduced fitness in the hybrids and/or F2 progeny in a type of Bateson-Dobzhansky-Muller (BDM) incompatibility that leads to reproductive isolation. A beautifully characterized example of this is found in the marine copepod *Tigriopus,* where inter-population mating resulted in mitonuclear specific fitness declines in F2 offspring [6–11]. Hybridization experiments in other taxa have also revealed mitonuclear incompatibilities, including flies [12–17]*,* wasps [18, 19], plants [20], nematodes [21, 22], yeasts [23–26], mice [27], and mammalian cell lines [28–30]. This growing body of literature lends support to the idea that BDM-type mitonuclear incompatibilities can contribute to, and even initiate, speciation [31].

While the coevolution of mitochondrial and nuclear genomes does occur, the unique biology of mitochondria complicates our view of a species’ evolutionary history [32–38]. In most metazoans, uniparental inheritance, lack of recombination and high mutation rate of mtDNAs make it difficult to separate the effect of genetic drift from natural selection [39]. Phenotypes associated with mtDNAs will depend on mtDNA copy number as well as allele frequencies, which can fluctuate in cells in tissue and environment-specific ways. The dynamic nature of mitochondria results in selective degradation of mitochondrial components including mtDNAs through fission events, and the rescue of metabolically-deficient mitochondria through fusion events [40]. Perhaps the major challenge in identifying coevolved mitonuclear genotypes is the environmentally-sensitive nature of phenotypes that result from mitonuclear interactions. For instance, differences in diet and oxygen availability altered mitonuclear interactions in *Drosophila* [15, 41]. These three-way mtDNA × nDNA × environment interactions may explain why replacing mtDNAs within a species can lead to more pronounced effects than that of between species [13] and why it can be challenging to conclusively document mitonuclear coadaptation [36].

*Saccharomyces* yeasts offer advantages for studying mitonuclear coevolution. Haploid viability and growth in tightly controlled growth environments will expose recessive traits while controlling for gene(s) x environment interactions. *Saccharomyces* yeasts can produce energy through fermentation pathways and are viable in the absence of mtDNAs. This quality facilitates the transfer of mtDNAs between haploid cells without the need for backcrossing strategies [42]. When two cells containing mtDNAs are mated, the mtDNAs are biparentally inherited and undergo recombination, followed by rapid fixation of a single mitochondrial haplotype (mitotype) [43, 44]. Controlled laboratory experiments allowing for mitochondrial recombination revealed that reassorting naturally-occurring alleles from different mitochondrial loci could produce functional differences [45, 46] and provide the opportunity to investigate fitness effects of coadapted mitonuclear alleles at high resolution. Advanced molecular tools for exploring mitochondrial mechanisms [43, 47] and an expanded knowledge of population genetics in *Saccharomyces* [48] further promote yeast as an exciting mitonuclear model.

*Saccharomyces* yeasts have a highly structured population [49, 50] with limited outcrossing [44]. Mitochondrial recombination is an important consequence of yeast matings in nature [45, 46, 51, 52], indicating that beneficial mitochondrial alleles could be isolated and available for selection. This is most likely to occur when yeasts mate outside their local population, such as when transferred by insect and human vectors [53, 54] where hybridization with local yeasts can occur [55]. Most yeast cells are likely to experience a singular environment during their life span due to their non-motility and short replication time. In theory, this should limit potential conflict between mitonuclear interactions in different environments [32] and promote intergenomic coadaptation in local populations. As would be expected for the interruption of coadapted mtDNA and nuclear genomes, interspecific *Saccharomyces* hybrids demonstrated different temperature tolerances [56, 57] and respiratory growth [26] depending on which species’ mtDNA was present. Similarly, respiratory capabilities were dependent on the origin of mtDNA in cybrids containing the nuclear genome of one *Saccharomyces* species and the mtDNA of another [23, 24].

In this study, we further develop *Saccharomyces* as a model for studying mitonuclear epistasis. We present the largest mitonuclear strain collection to date, containing 225 unique mitonuclear genotypes that allows for direct testing of mitonuclear epistasis. We observed that environmentally-sensitive mitonuclear interactions explain a substantial portion of phenotypic variation, consistent with our previous studies on smaller numbers of mitonuclear strains [45, 58]. We also found evidence for mitonuclear coadaptation, suggesting that selection for specific mtDNA-nuclear interactions has occurred repeatedly during the population expansion of yeast.

## Results

### A mitonuclear strain collection allows for direct assessment of mitonuclear interactions

**Fig. 1:**
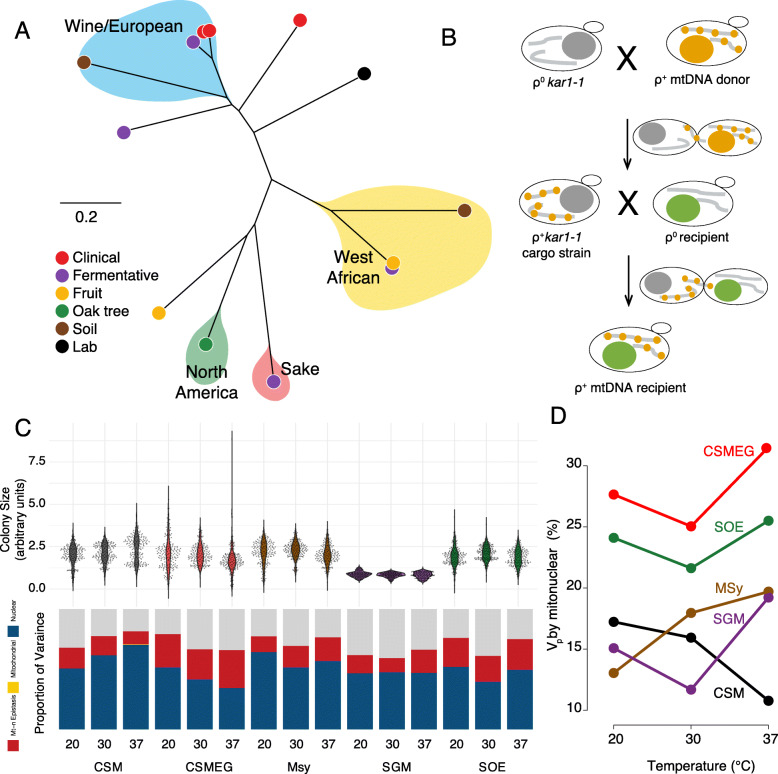
Mitonuclear interactions contribute to phenotypic differences in *S. cerevisiae****.*** **a**. Neighbor-joining tree of 13 (of 15) yeast isolates used as parental strains for the mitonuclear collection. Genetic distances, clades and ecological origins are from [49]. Lower resolution genetic data [54] places one additional parental strains as wine/European and a second as mosaic. See Table S1 for strain details. **b**. Mating strategy for creation of the mitonuclear strain collection. mtDNAs were passaged from 15 parental strains through *kar1–1* cargo strains to recipient strains to create 225 unique mtDNA-nuclear genotypes. Selection and screening regimes explained in methods. **c**. Fitness measures (colony sizes) of the mitonuclear strain collection in all conditions, including standard laboratory media containing fermentable (CSM) and non-fermentable (CSMEG) carbon sources, and media simulating ecological habitats including synthetic grape must (SGM), maple sap (MSy) and synthetic oak exudate (SOE) at three temperatures (20, 30, and 37 °C), are shown as violin plots. Proportion of variances due to mtDNA (yellow), nuclear genetic background (blue), mitonuclear interactions (red) and residual (gray) are indicated in bar graphs. **d**. Temperature-dependent mitonuclear effects. In most media, mitonuclear interactions contribute to a higher proportion of V_p_ under low or high temperature stress

To explore how mitonuclear interactions contribute to phenotypic differences in *S. cerevisiae*, we chose 15 isolates that were available as haploid derivatives [59]. These isolates represented wild and domesticated yeasts with mosaic (*n* = 5), wine/European (n = 5), West African (*n* = 3), Sake/Asian (*n* = 1), and North American (n = 1) lineages (Fig. 1a, Table S1). Phenotype and SNP-derived genetic distance correlations for these strains were modestly correlated (Pearson’s r = 0.2 to 0.5, Fig. S1), consistent with previous analyses of much larger numbers of yeast isolates (Pearson’s r = 0.3 to 0.6) [50, 54, 60–62]. The mitochondrial genomes from these 15 isolates are highly polymorphic with unique allelic profiles across their coding sequences (Table S2), with the exception of 2 wine/European strains (YJM981 and YJM975) that have identical mitochondrial coding sequences. The nucleotide diversity for these 15 mitochondrial coding sequences is slightly higher than found across a comparison of 353 mtDNAs (0.01 (Table S3) vs. 0.0085 [52] with the majority of sequence differences found in *COX1, COX2* and *VAR1*. These mtDNAs did not present evidence of gene rearrangements or interspecific introgressions as have been sometimes observed [51, 52]. While these selected isolates do not necessarily represent all the genetic diversity that describes *S. cerevisiae* populations [49, 52], they do capture a substantial amount of the genetic complexity leading to trait variation.

In populations, detection of epistatic interactions between different loci is limited to those with large effect as most statistical approaches are hampered by multiple testing and power issues [63]. Here, we created a mitonuclear strain collection by precisely exchanging mtDNAs between 15 parental strains to create 225 unique mitochondrial-nuclear genome combinations (15 mtDNAs × 15 nuclear genomes) (Fig. 1b). The mitonuclear strains (including biological replicates of each genotype and reconstructed parental genotypes) were grown in 15 different conditions. These environments were 5 different media (oak exudate (SOE), synthetic grape must (SGM), maple sap media (MSy) and lab media containing glucose (CSM) and ethanol/glycerol (CSMEG), each grown at three temperatures (20, 30, and 37 °C), resulting in 67,727 fitness estimates. Distribution of colony sizes varied among conditions with the widest range of phenotypes seen in media requiring mitochondrial respiration for growth under high temperature stress (CSMEG 37 °C) (Fig. 1c).

The balanced design of the mitonuclear strain collection allows for direct testing of mitonuclear epistasis. Highly significant mitonuclear interaction terms were observed in each condition (ANOVA on the random effect model y_ij_ = μ ~ mt_i_ + n_j_ + (mt × n)_ij_ + ε_ij_, *P* < 0.0001, Table S4) and could explain 10.8 to 31.5% of the total phenotypic variances (Fig. 1c, Table S5). Mitonuclear interactions explained the highest proportion of phenotypic variances in media requiring mitochondrial respiration (CSMEG), consistent with the idea that physically interacting structural subunits of the oxidative phosphorylation machinery would be particularly sensitive to genetic variation that altered the efficacy of ATP production. Growth in high and low temperatures generally increased the effects of mitonuclear interactions (Fig. 1d) consistent with the known roles of mitochondria in stress responses [64]. Mitonuclear interactions were sensitive to media and temperature (ANOVAs for mt × n × media or mt × n × temperature) (Table S6) and indicates that epistatic interactions different functional mitochondrial and nuclear allelic combinations in different environmental conditions. No independent mitochondrial effects were observed in this collection (*P* = 1, Table S4), highlighting the importance of inter-genomic communication for mitochondrial functions. Mitonuclear epistasis was widespread throughout this population; ANOVA tests performed on each combination of 4 mitonuclear genotypes (2 original and the 2 synthetic genotypes following an exchange of mtDNAs) revealed significant mitonuclear epistasis in 318 of 585 tests (54%) (Table S8).

Strains containing synthetic mitonuclear genotypes resulted in growth phenotypes outside the ranges of those observed in the parental strains (Table S7, Fig. S2), providing both fitness advantages and disadvantages. All strains harboring synthetic mitonuclear combinations supported growth on non-fermentable carbons and thus, did not contain the strong mitonuclear incompatibilities preventing mitochondrial respiration that has been observed in some interspecific cybrids containing the nucleus of one species and the mtDNA of another [24–26, 65]. Still, our strain collection reveals that intraspecific mitonuclear interactions provide a substantial phenotypic landscape upon which selection can act that may ultimately lead to interspecific BDM-type incompatibilities.

### Mitonuclear coadaptation revealed in ecologically relevant environments

**Fig. 2:**
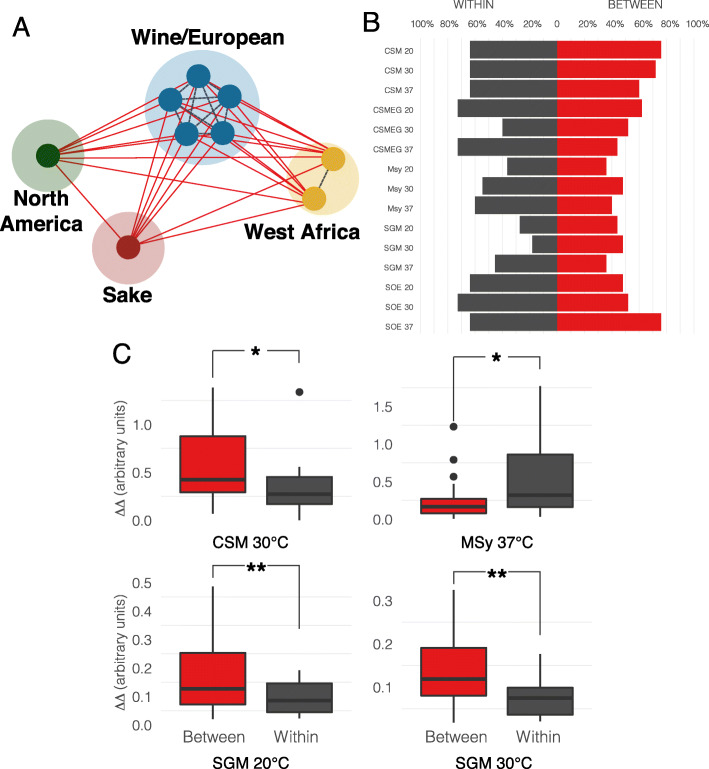
Genetic distance influences the effect of mitonuclear interactions. **a**. All possible mtDNA exchanges among strains with clean (non-mosaic) lineages. Significant mitonuclear epistasis was expected to result from mtDNA exchange between two strains from different subpopulations (red solid lines), but not from mtDNA exchanges within a subpopulation (dark gray dotted lines). **b**. Frequencies of significant mitonuclear epistasis within and between clades. (see Table S9 for exact numbers). **c**. Mitonuclear effect sizes were significantly different in 4 conditions. Between clade mtDNA exchanges (red) often resulted in stronger epistatic effects (ΔΔ) than within clade exchanges (dark gray). ΔΔ = ∣(Δ_nDNA_^i^_/mtDNA_^i^_→nDNA_^i^_/mtDNA_^j^) – (Δ_nDNA_^j^_/mtDNA_^i^_→ nDNA_^j^_/mtDNA_^j^) ∣; * *P* < 0.05, ** *P* < 0.005

Selection for mitonuclear interactions could contribute to phenotypic differences between different clades of yeast. We reasoned that if selection for specific mitonuclear allele pairings occurred during the initial expansions of *S. cerevisiae*, exchanging mtDNAs between strains within the same clade should have less of an effect than between clades. Our collection contained five strains from a Wine/European clade, two from a West African clade and single representatives from Sake and North American clades, providing 11 within- and 25 between-clade mtDNA exchanges (Fig. 2a)*.* (One additional West African strain was highly flocculant and was not included here.) For each exchange, we tested whether mitonuclear interactions explained growth differences between strains harboring original (nDNA^i^/mtDNA^i^ and nDNA^j^/mtDNA^j^) and synthetic (nDNA^i^/mtDNA^j^ and nDNA^i^/mtDNA^j^) mitonuclear combinations using fixed effects two-way ANOVAs (Table S8). Within clade mtDNAs exchanges were just as likely to show significant mitonuclear epistasis as between clade exchanges in most media conditions (*P* > 0.05 for Χ^2^ tests, Table S9**,** Fig. 2b**)**. However, mitonuclear interactions produced larger effect sizes (measured as the absolute differences between the changes in growth in two strains following the exchange of their mtDNAs) when mtDNAs were exchanged between clades in 3 (of 15) conditions (Fig. 2c). This is consistent with a model where populations maintain separate mitonuclear alleles that diverge over time. In one environment, mitonuclear effects were greater in the within-clade mtDNA exchanges (and were not different in the remaining ten environments). Taken together, these results demonstrate that natural genetic variation in mitonuclear interactions will alter phenotypes but genetic distance alone cannot necessarily predict the effects of these interactions in all environments.

Previously, we showed that a subset of the strains harboring original mitonuclear combinations conferred higher growth rates than strains with synthetic combinations at elevated temperature and under respiratory conditions [58]. In this larger survey of mitonuclear combinations, strains with their original mtDNAs did not show significant fitness advantages over introduced mtDNAs, except in SGM media at 30 °C (Fig. S2). Because fitness effects of mitonuclear interactions are sensitive to environmental conditions (Table S6) and because any selection for optimal mitonuclear interactions would have occurred in ecologically relevant conditions, it is perhaps not meaningful to compare average fitness effects for all strains in the same environment. We reasoned that the fitness advantage of original mitonuclear pairings observed in SGM media might be explained by the overrepresentation of strains isolated from fermentation sources (5 of the 15 parental strains) whose mitonuclear genomes were coadapted for an alcoholic fermentation niche.

**Fig. 3:**
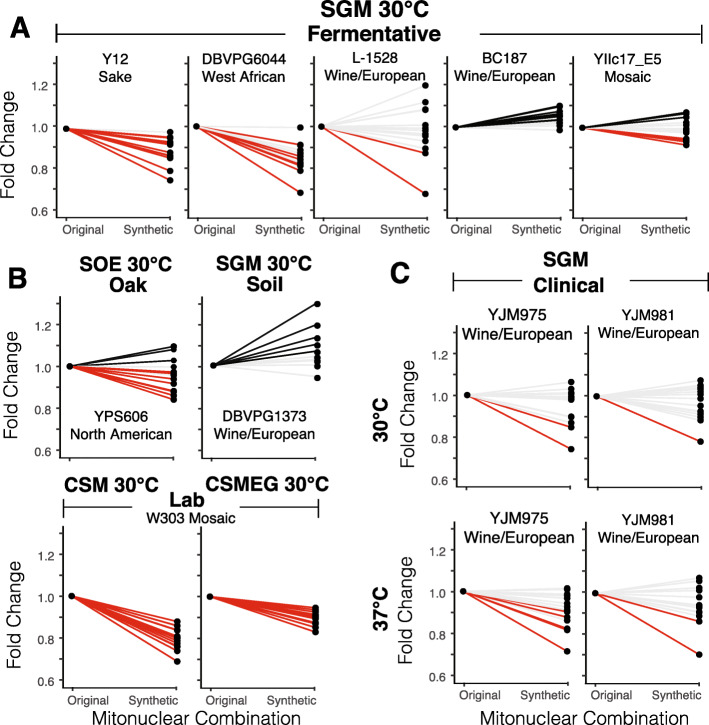
Evidence for mitonuclear coadaptation. For each nuclear background, growth rates of strains containing parental and synthetic mitonuclear genotypes were compared using one-way ANOVAs. Genotypes are connected by colored lines to indicate when the original, coadapted genotype showed fitness advantages (red) or disadvantages (black) as compared to the synthetic genotype (P < 0.05). Non-significant growth differences are shown by light gray lines. Growth rates were normalized to the parental mitonuclear genotype for presentation purposes. Strains were tested in ecologically-relevant media at 30 °C including **a**. fermentation isolates in SGM; **b**. oak, soil, and laboratory isolates in SOE, SGM and CSM/CSMEG, respectively; **C**. clinical isolates belonging to the Wine/European clade in SGM at 30 °C and 37 °C

To determine if the original mitonuclear genome pairings were coadapted to isolation habitats, we paired the parental strains with media that best resembled isolation habitats and asked whether each nuclear background preferred its own (coadapted) or foreign mtDNA (Fig. 3). Considering fermentation isolates in a synthetic grape must media, we found that 3 of 5 coadapted mitonuclear combinations provided growth advantages. Strains with nuclear backgrounds from Sake (Y12), West African (DBVPG6044) and wine/European (L-1528) clades had higher growth rates when paired with their original mtDNAs than with any foreign mtDNAs (Fig. 3a). Synthetic mitonuclear combinations in strains derived from the nuclear background of the wine isolate BC187 showed slight but statistically significant advantages over the coadapted mitonuclear combination. Interestingly, no preference was seen for the coadapted mitonuclear combination when the nuclear background originated from a wine strain with mosaic ancestry (YIIc17_E5). In this case, synthetic mitonuclear genotypes were just as likely to show fitness advantages as disadvantages. It is possible that a recent hybridization event interrupted ancestral coadapted mitonuclear complexes in this mosaic parental strain.

Beneficial coadapted mitonuclear genome combinations could also be observed in other strains. The original mitonuclear genotype derived from an oak (*Quercus*) isolate (YPS606) grew better when paired with its own mtDNA than with 8 others when tested in an oak exudate medium (Fig. 3b). The experimental model strain W303, maintained under laboratory conditions since the 1970s [66], grew better with its own mtDNA than with any other mtDNA when tested in common laboratory media CSM and CSMEG (Fig. 3b). The original mitonuclear genotypes from 2 clinical isolates of Wine/European ancestries were more likely to show growth benefits when grown at high temperatures in grape must media (Fig. 3c). The original mitonuclear genotype from a soil isolate of wine/European ancestry (DBVPG1373) was less likely to show fitness advantages in grape must media, suggesting that this isolate may have undergone different ecological pressures from the fermentation isolates of the same clade. Given the reticulate ancestries of *S. cerevisiae* mtDNAs [50–52, 67], mitochondrial SNPs are not necessarily in linkage disequilibrium. In order to identify mitochondrial SNPs that conferred the observed coadapted phenotypes, we performed association tests (Table S10) for each set of 15 strains with a common nuclear genotype in the conditions shown in Fig. 3. No significant associations were observed, potentially because of low statistical power and relatively small phenotypic differences. Taken together, these data suggest that selection for mitonuclear interactions is largely strain specific and dependent on environmental conditions.

## Discussion

Our study provides insight into the coevolution of mitochondrial-nuclear genotypes in *S. cerevisiae* yeasts. Ancestral relationships were less likely to predict when coadapted mitonuclear genotypes would provide overall growth benefits, as noted by the observation that mitonuclear epistasis was just as likely to be observed when mtDNAs were exchanged between or within clades. However, between-clade mtDNA exchanges did result in larger epistatic effects (ΔΔ) in a variety of media, hinting at the idea of ancestral selection for mitonuclear alleles. Our data showed that media, particularly when matched to an isolation source, was a better predictor of when a coadapted mitonuclear genotype would provide growth advantages. For examples, the original mitonuclear genotypes resulted in higher growth rates in three of five wine isolates when assayed in a grape must media and in an oak tree isolate when assayed in a synthetic oak exudate medium. It is difficult to know if these laboratory conditions mimicked the environments these yeasts experienced in nature as relatively little is known about the ecological niches that yeasts occupy [68, 69]. Still, it is likely that as these yeasts entered new environments, ancestral mitonuclear interactions began to be lost as new beneficial mitonuclear alleles were selected for. Consistent with this idea, two clinical isolates of wine/European ancestries showed an increase in the preference for their coadapted mitonuclear combinations when grown at elevated temperatures (representing the new ecological niche) in a grape-must (ancestral) media. Rapid selection for mitonuclear alleles is also supported by the observation that a lab strain preferred its original mtDNA when assayed in common laboratory media. Interestingly, in wild *S. cerevisiae* yeasts, strains from mixed clades with diverse ecological and geographic origins had lower mitochondrial coding sequence variation than in other populations [52]. This is in contrast to what would be expected if selection for different mitonuclear alleles were important for adaptation to new environments. It is possible that the admixtures were too recent to detect mitochondrial nucleotide diversity obtained through mitochondrial recombination.

By precisely replacing mtDNAs between 15 *S. cerevisiae* isolates, we could distinguish phenotypic effects of mitonuclear interactions from other possible genetic interactions. Consistent with the fact that mtDNAs do not function alone and in line with studies in invertebrate models [14], we did not observe independent mtDNA effects on phenotypes (Table S4) unless we focused on small subsets of our strain collection (Table S8). This highlights the need to consider epistatic effects when investigating mitochondrial physiology. Our results support the emerging recognition that environmentally-sensitive mitonuclear epistasis leading to phenotypic variation is a universal feature in biology. The methodologies used to create these strains, including the generation of rho^0^ strains and karyogamy-deficient matings, can be mutagenic [70, 71] and thus, we cannot guarantee that this mitonuclear strain collection is absent of mutations. It is unlikely that hidden mutations confounded our analyses. No phenotypic differences between the haploid parental strains and those with recreated parental genotypes were observed for the coadapted strains shown in Fig. 3 (ANOVA, *P* > 0.05), nor in an earlier study [58]. In this study, we included independently derived biological replicates for each genotype. In theory, strain-specific mutations introduced during strain construction should increase phenotypic variance within a genotype and decrease the ability to detect mitonuclear epistasis. Still, we detected significant mitonuclear epistasis in 54% of independent epistasis tests. Widespread occurrence of mitonuclear interactions is also supported by other studies in *Saccharomyces*. A large panel of iso-nuclear heterozygous *S. cerevisiae* yeasts revealed that respiratory and non-respiratory growth phenotypes were dependent on nuclear and mitochondrial backgrounds [72]. Most likely, dominant nuclear alleles contributed to the mitonuclear phenotypes in these diploid strains. This should facilitate selection of mitonuclear allele combinations in natural populations. Consistent with selection for particular mitochondrial and nuclear interacting loci, the recombinant mitochondrial haplotypes in naturally occurring *S. paradoxus* hybrids carried uneven contributions of the parental mtDNAs and influenced phenotypes [46].

Identifying the exact loci underlying mitonuclear interactions is an obvious future goal. This study highlights that mapping efforts will need to consider that coadapted mitonuclear loci are likely strain and niche specific. Most analysis of yeast mtDNAs have focused on coding sequences [51, 52, 73]. We did not find associations between mitochondrial coding SNPs and growth phenotypes in environments revealing coadapted mitonuclear genotypes. While higher statistical power and stronger phenotypes would be beneficial for association-based mapping, it is important to recognize that mitonuclear interactions may not be restricted to coding sequences. Exchanging mtDNAs between strains whose mitotypes have identical mitochondrial coding sequences but variable intergenic sequence produced significant mitonuclear interactions in some environments (Table S8). The parental strains are known to have different mtDNA copy numbers, in addition to different mtDNA-dependent replicative lifespans [74], raising the possibility that intergenic mitochondrial sequences that influence mtDNA replication or copy number may be important components of mitonuclear epistasis.

## Conclusions

Genetic interactions between mtDNA and nuclear genomes, especially under temperature stresses, contribute to phenotypic variation within and between populations of *S. cerevisiae* yeasts. Statistically significant mitonuclear interactions were observed when exchanging mtDNAs between closely related strains, although the overall mitonuclear effect sizes were largest when mtDNAs were exchanged between clades (subpopulations) of *S. cerevisiae*. Coadapted mtDNA-nuclear genome pairings provided fitness advantages in media emulating isolation habitats, but did not necessarily provide fitness advantages in other environments. Our study provides a substantial resource for further research into mitonuclear coadaptations.

## Methods

### Strains

Yeast strains used or created for this study are provided (Table S1). The parental yeast strains [59] were purchased from the National Collection of Yeast Cultures (SGRP strains).

### Media

Media included the standard laboratory media YPD (10 g/L yeast extract, 20 g/L peptone, 20 g/L glucose). YPEG (10 g/L yeast extract, 20 g/L peptone, 30 mL/L ethanol, 30 mL/L glycerol), Complete synthetic media (CSM) (800 mg/L CSM premix (Sunrise Science), 6.7 g/L yeast nitrogen base without amino acids (YNB w/o AA), 20 g/L glucose), and CSMEG (CSM containing 30 mL/L ethanol and 30 mL/L glycerol instead of glucose). CSM media lacking specific amino acids were prepared by replacing CSM premix with those formulated to omit specific amino acid drop out mixes (CSM-Adenine, CSM-Uracil, CSM-Arginine), as recommended by the manufacturer (Sunrise Science). Additional media included SOE, a synthetic oak exudate [75] containing 1 g/L yeast extract, 1.5 g/L peptone, 10 g/L sucrose, 5 g/L fructose, 5 g/L glucose). Media emulating maple sap (MSy) [76] contained 1.75 g/L YNB w/o AA, 800 mg/L CSM premix, 0.5 g/L allantoin, and a locally-produced maple syrup added to a final concentration of 2° Brix.

Synthetic Grape Must (SGM) [77, 78] contained 125 g/L glucose, 125 g/L fructose, and 460 mg/L ammonium chloride, supplemented with vitamin, mineral salts, and amino acid mixes. To mimic the normal concentrations of these acids commonly found in must at grape maturity, 3 g/L malic acid, 3 g/L tartaric acid, and 0.3 g/L citric acid, as well as anaerobic factors (15 mg/L ergosterol and 5 mg/L sodium oleate dissolved in (1,1,v/v) ethanol: Tween 80) were added to media, then the pH was adjusted to 3.0 using NaOH. The vitamin mix used in SGM media contained 20 mg/L myo-inositol, 2 mg/L nicotinic acid, 1.5 mg/L calcium pantothenate, 0.25 mg/L thiamine HCl, 0.25 mg/L pyridoxine HCl, 0.003 mg/L biotin, and mineral salts (750 mg/L KH_2_PO_4_, 500 mg/L K_2_SO_4_, 250 mg/L MgSO_4_ · 7H_2_O, 55 mg/L CaCl_2_ · 2H_2_O, 200 mg/L NaCl, 4 mg/L ZnSO_4_, 1 mg/L CuSO_4_ ·5H_2_O, 1 mg/L KI, 0.4 mg/L CoCl_2_·6H_2_O, 1 mg/L H_3_BO_3_, 1 mg/L NaMoO_4_·2H_2_O). The amino acid mix used in SGM media contained 19 amino acids (612.6 mg/L L-proline, 505.3 mg/L L-glutamine, 374.4 mg/L L-arginine, 179.3 mg/L L-tryptophan, 145.3 mg/L L-alanine, 120.4 mg/L L-glutamic acid, 78.5 mg/L L-serine, 759.2 mg/L L-threonine, 48.4 mg/L L-leucine, 44.5 mg/L L-aspartic acid, 44.5 mg/L L-valine, 37.9 mg/L L-phenylalanine, 32.7 mg/L L-isoleucine, 32.7 mg/L L-histidine, 31.4 mg/L L-methionine, 18.3 mg/L L-tyrosine, 18.3 mg/L L-glycine, 17.0 mg/L lysine, 13.1 mg/L L-cysteine,35 mg/L uracil, 35 mg/L adenine hemisulfate).

For solid media, agar was added to 2% prior to autoclaving. Due to agar disintegration in low pH, 4% agar solution was prepared separately and added to SGM mixture (1,1, v/v) after autoclaving.

### Creation of a mitonuclear strain collection

To generate strains with 225 unique mitonuclear genotypes, mtDNAs were transferred between 15 divergent *S. cerevisiae* isolates using karyogamy-deficient matings (Fig. 1b). Each parental strain (*MATa ura3* ρ^+^) was mated to NAB32 (*MAT*α *kar1–1 ade2 arg8* ρ^0^*)* on solid YPD media. When zygotes were visible under a compound microscope (2–6 h), mating mixtures were diluted into YPD liquid media and incubated for 2 h to promote cell division. Cell mixtures were plated for single colonies on media that selected against the mtDNA donor strain (CSM–URA). Colonies were printed to selective media (YPEG, CSM–ADE and CSM–ARG) and mitochondrial cargo strains were identified as respiring colonies with auxotrophies of the *kar1–1* recipient strain. Cargo strains were mated with rho^0^ derivatives of each parental strain, followed by selecting and screening for respiring haploids with the genotype of the parental strains. At least 2 biological replicates for each mitonuclear genotype were isolated from independent matings. As controls, each of the original mtDNAs were reintroduced to the rho^0^ parental strains to recreate parental mitonuclear combinations. Rho^0^ strains were generated using ethidium bromide [79]. Strain names and genotypes for the complete 15 × 15 mitonuclear strain collection are found in Table S1.

### Phenotyping

Strains were phenotyped by spotting cells in high density arrays using a BM3-BC colony processing robot (S&P Robotics) using a 4 × 8 block design. Arrays were printed from YPD to test media, acclimated for 2 days at 30 °C, reprinted to test media (CSM, CSMEG, MSy, SGM, and SOE) and incubated at 20°, 30° and 37 °C. Strains with nuclear backgrounds strains SK1 and 322136S were omitted as they were flocculant. Arrays were photographed over 96 h at 18 time points. Colony sizes from each image were determined using gitter [80] and were fed through a custom R script pipeline (modified from [45]). Colony spots that failed circularity measures were omitted from further analyses. Colonies from the two outermost rings of each plate were removed to avoid edge effects. Colony spots were used to fit growth curves using logistic regression and outliers were omitted. Fitness parameters (minimum and maximum colony size) were estimated from logistic growth curves and normalized to a reference strain (DAU2) included on each array. To correct for unequal numbers of technical replicates for strains with a shared genotype (i.e. biological replicates), a random subset of technical replicates was selected with equal numbers of each biological replicate. The difference between maximum and minimum colony size was used as a proxy for fitness.

### Statistical analysis

Statistical analyses were performed with the lme4 package in R (version 3.6.0). Eight mitochondrially-encoded genes were obtained from available mitochondrial sequences [50, 81–83] (Table S2). Alignments and identification of SNPs and nucleotide diversities were performed using Geneious version 2020.5 [79].

The significance of nuclear, mitochondrial genetic components, environments and their interactions were tested using random effect models (lmer) by comparing the full model n + mt + e + (n × e) + (n × e) + (mt × n) + (mt × n × e) with a model lacking the evaluated term. Within individual environments, random effects models were used to determine the significance of nuclear, mitochondrial genotypes and mitonuclear interactions. Variance component analysis was performed in each condition (VarCorr), in which the contributions of each genetic term to phenotypic variance were estimated from the full model: n + mt + (mt × n).

To estimate the frequency of mitonuclear epistasis when exchanging mtDNA strains from the same or two different subpopulations, two-way ANOVAs were used to test the significance of mitonuclear interactions among each mtDNA exchange such that each test included 4 genotypes: two parental mitonuclear genotypes and two synthetic genotypes derived from the exchange of mtDNAs between the parental strains. The mitonuclear effect size in these exchanges was determined as the absolute differences between the change in growth for each nuclear genotype (ΔΔ = ∣(Δ_nDNA_^i^_/mtDNA_^i^_→nDNA_^i^_/mtDNA_^j^) – (Δ_nDNA_^j^_/mtDNA_^i^_→ nDNA_^j^_/mtDNA_^j^) ∣).

To test the effect of disrupting naturally occurring mitonuclear combinations, one-way ANOVA was used to test for significant difference in fitness between original strains and 195 synthetic strains in each condition. Within each nuclear background, the significance in fitness difference between the strain with coadapted mitonuclear combinations and each of its 14 synthetic derivatives was investigated using a fixed effect model.

We performed association tests between 198 mitochondrial SNPs (Table S2) and the phenotypes listed in Fig. 3 using the 15 strains sharing a common nuclear background in each media type. ANOVAs were conducted in R using a simple linear model (phenotype ~ SNP_i__) and Bonferroni corrections were applied to determine significance thresholds (*P* < 0.00025).

## Supplementary information

**Additional file 1:** **Table S1.** Strain table. **Table S2.** SNP table for mitochondrial coding sequences for the 15 parental strains. Allzle numbering is arbitrary. **Table S3.** Mitochondrial coding sequence statistics. **Table S4.** Mitochondrial × nuclear interactions (mt × n) within single environments. To determine the significance of each term, ANOVAs comparing the full model with a model lacking the indicated term were evaluated. Each factor was treated as a random effect. Full model: n + mt + (mt × n). **Table S5.** Phenotypic variance analysis to show the amount of variance contributed by mitochondrial, nuclear background, and mitonuclear interactions in all growth conditions. **Table S6.** Mitochondrial × nuclear × environment interactions (mt × n × e). To determine the significance of each term, ANOVAs comparing the full model with a model lacking the indicated term were evaluated. Each factor was treated as a random effect. Full model: n + mt + e + (mt × n) + (n × e) + (mt × e) + (mt × n × e). **Table S7.** Phenotypic variance of strains with original, synthetic, and all possible mitonuclear combinations across all growth conditions. Student’s t-test were performed to determine whether the difference among strains with original and synthetic combinations is statistically significant. **Table S8.** Fixed effect model, two-way ANOVA table showing mt × n interactions for each mtDNA exchange between two strains. The *P* values for genetic terms (nuclear, mitochondrial, mitonuclear epistasis), type of epistasis, and estimated strength of epistasis were provided for each pairwise mtDNA exchange. **Table S9**. Numbers of significant mitonuclear tests following mtDNA exchanges within and between clades. **Table S10**. Mitochondrial genotype-phenotype association tests.

**Additional file 2: Fig. S1** Nuclear genotype-phenotype correlations. Growth differences between 12 *S. cerevisiae* parental strains were plotted against nuclear genetic distances in 5 different media (CSM, CSMEG, MSy, SGM, and SOE) at 3 temperatures (20 °C, 30 °C, and 37 °C) (see Methods for descriptions of media and of fitness measurements). Genetic distance estimates (percentage values) based on pairwise SNP differences in the alignments were obtained from [49]. Pearson’s correlation values are shown. Regression lines for significant correlations are shown in red. Similar correlations are observed using the lower resolution genetic distances from [54] and phenotyping data for all 15 parental strains (not shown).

**Additional file 3 Fig. S2** Coadapted vs. synthetic mitonuclear combinations. Average growth rates of strains with original (yellow) or synthetic (gray) mitonuclear genome combinations across all different growth conditions (Student t-test). Coadapted mitonuclear combinations did not provide overall growth advantages in all media. Significance codes: ** < 0.01, * < 0.05.

## Data Availability

Strains and fitness measures for each strain are available from the corresponding author on reasonable request. Custom R scripts used to fit growth curves and estimate growth rate are available at https://github.com/Tuc-Nguyen/HLF-Robot-Image-Analysis-2.1.

## Abbreviations

BDM: Bateson–Dobzhansky–Muller
YNB w/o AA: Yeast nitrogen base without amino acids
YPD: Fermentable, rich media
YPEG: Non-fermentable, rich media
CSM: Fermentable, defined complete synthetic media
CSMEG: Non-fermentable, defined complete synthetic media
SGM: Synthetic Grape Must Media
MSy: Maple Syrup Media
SOE: Synthetic Oak Exudate Media
mtDNA: Mitochondrial DNA
nDNA: Nuclear DNA
mt × n: Mitonuclear interactions
e: Environmental effect
V_p_: Phenotypic Variance
