# Supplementary figures and images for: Mitochondrial-nuclear coadaptation revealed through mtDNA replacements in Saccharomyces cerevisiae
Source: BMC Evol Biol. 2020 Sep 25;20:128. doi: 10.1186/s12862-020-01685-6 (PMC7517635; doi:10.1186/s12862-020-01685-6)

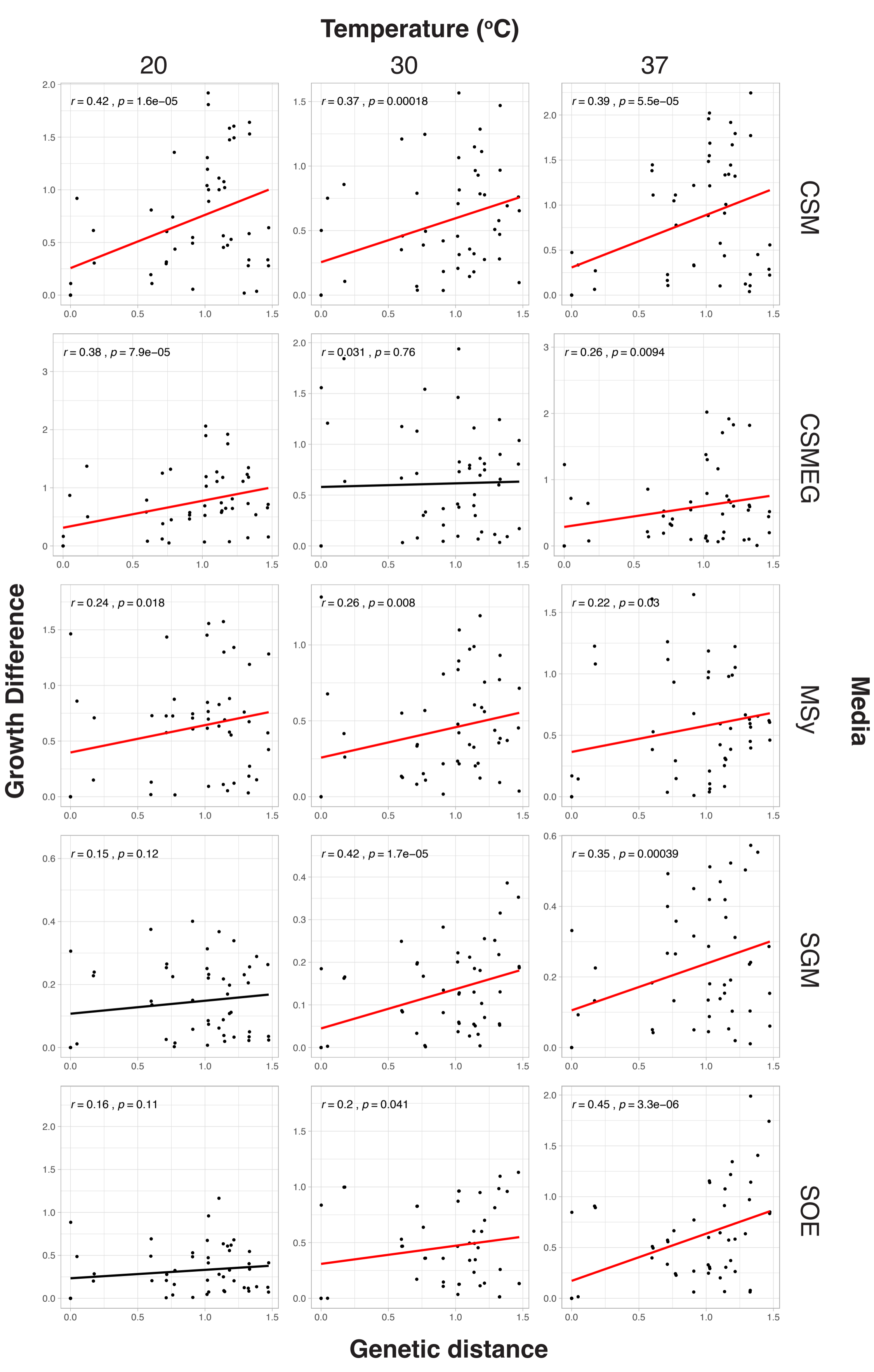

Supplement: Supplementary file 2 — Additional file 2: Fig. S1 Nuclear genotype-phenotype correlations. Growth differences between 12 S. cerevisiae parental strains were plotted against nuclear genetic distances in 5 different media (CSM, CSMEG, MSy, SGM, and SOE) at 3 temperatures (20 °C, 30 °C, and 37 °C) (see Methods for descriptions of media and of fitness measurements). Genetic distance estimates (percentage values) based on pairwise SNP differences in the alignments were obtained from [49]. Pearson’s correlation values are shown. Regression lines for significant correlations are shown in red. Similar correlations are observed using the lower resolution genetic distances from [54] and phenotyping data for all 15 parental strains (not shown). [file 12862_2020_1685_MOESM2_ESM.pdf]

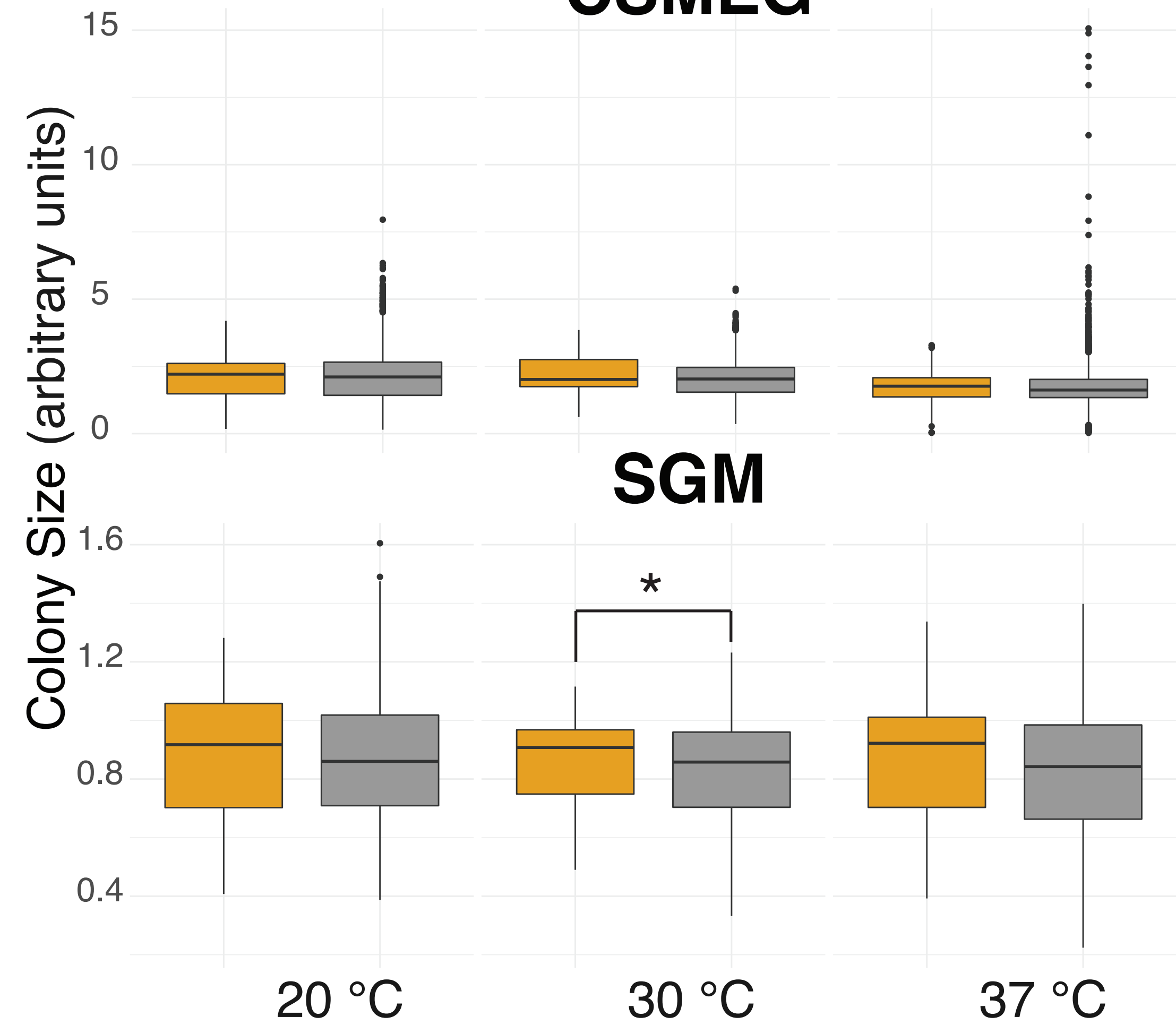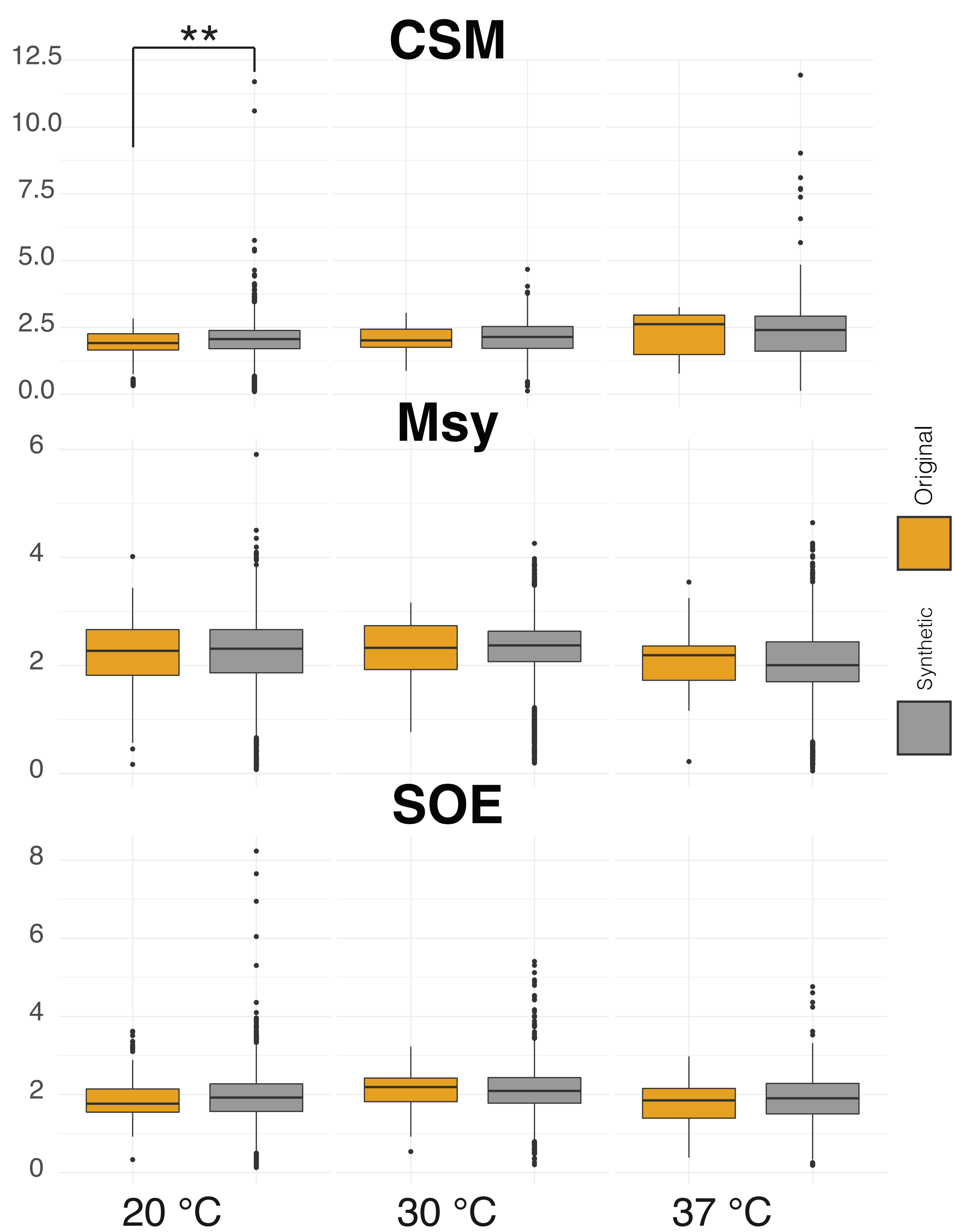

Supplement: Supplementary file 3 — Additional file 3 Fig. S2 Coadapted vs. synthetic mitonuclear combinations. Average growth rates of strains with original (yellow) or synthetic (gray) mitonuclear genome combinations across all different growth conditions (Student t-test). Coadapted mitonuclear combinations did not provide overall growth advantages in all media. Significance codes: ** < 0.01, * < 0.05. [file 12862_2020_1685_MOESM3_ESM.pdf]
